# FOXP3 inhibits MYC expression via regulating miR‐198 and influences cell viability, proliferation and cell apoptosis in HepG2

**DOI:** 10.1002/cam4.1780

**Published:** 2018-10-30

**Authors:** Xiaohui Duan, Bo Jiang, Jianhui Yang, Lixue Zhou, Bingzhang Tian, Xianhai Mao

**Affiliations:** ^1^ Department of Hepatobiliary Surgery/Research Laboratory of Hepatobiliary Tumor Hunan Provincial People's Hospital Changsha China

**Keywords:** FOXP3, liver neoplasms, miR‐198, MYC

## Abstract

**Objective:**

Our study aimed to explore the effects of FOXP3 expression on liver neoplasms cells and to further investigate the relationship between FOXP3 and proto‐oncogene MYC.

**Methods:**

QRT‐PCR was used for assessment of FOXP3 expression in liver neoplasms tissues and para‐carcinoma tissues. The effects of FOXP3 on cell viability were determined by CCK8 assay, clone formation experiment, and flow cytometry. For miRNA selection, chips were used to figure out the differentially expressed miRNAs in FOXP3‐overexpressing HepG2 cells. The result was followed by bioinformatics prediction to screen the possible MYC‐targeted miRNAs, and it was examined by dual luciferase assay and ChIP assay. The expression levels of MYC protein and apoptosis‐associated proteins (bcl2 and bax) were measured by Western blot assay.

**Results:**

It showed an under‐regulated expression of FOXP3 in liver neoplasm tissues from qRT‐PCR results. Overexpression of FOXP3 contributed to cell apoptosis as well as suppressed tumor cells’ proliferation. MiR‐198 was detected to be highly expressed in FOXP3‐overexpressing HepG2 cells. FOXP3 regulated the transcription level of miR‐198 by binding to its promoter sequence and overexpressed miR‐198 could suppress tumor cells’ proliferation and promote cell apoptosis. There existed targeted relationship between miR‐198 and MYC gene. MiR‐198 inhibited cancer by suppressing the expression of MYC in liver neoplasm.

**Conclusion:**

FOXP3 up‐regulated miR‐198 expression by binding to its promoter sequence specifically, while miR‐198 inhibited proto‐oncogene MYC via targeted relationship. High level of miR‐198 contributed to the apoptosis of tumor cells and suppressed cell viability meanwhile.

## 1 INTRODUCTION

Liver neoplasm is the fifth most common cancer in the world and has caused over 27 000 deaths in 2016 according to the National Cancer Society,^1^ and there has been a rising trend in incidence of liver neoplasm since 1975.^2^ Although plenty of physical, chemical, and biological treating methods have been developed for liver neoplasm such as resection, immune treatment, ablation, embolization chemotherapy, and targeted medicine, the greatly limited liver transplantation is still the only reliable way for patients.^3^ Therefore, it is an urge to develop more specific and efficient methods for clinical treating.

MicroRNAs (miRNAs) are small noncoding RNAs that widely arise in animals, plants, and even viruses. MiRNAs can suppress the translation of mRNAs by binding to the 3′‐UTR (untranslated region) of specific mRNAs.^4^ MicroRNA‐198 (miR‐198) is microRNA consist of 22 bases, known to have the function of inhibiting cell multiplication, migration, invasion, and promoting cell apoptosis in most tumor cells.^5^ There are a few researches revealing the connection between miR‐198 and liver neoplasm. Elfimova et al^6^ find that miR‐198 overexpression significantly reduced cell growth in Pop 10 hepatoma cells. Tan et al^7^ further investigate the underlying mechanism of miR‐198 inhibition and note the target relationship between HGF/c‐MET pathway and miR‐198.

Forkhead box P3 (FOXP3) protein is a transcription factor belonging to the forkhead/winged helix family, coded by the FOXP3 gene located on human chromosome X. FOXP3 exists in almost all of the mammalian somatic cells and has especially high expression in some of the T cells, which means an important role in immune regulation.^8^ Notably, recent researches have revealed that FOXP3 is not only the master regulator in T cells but also works as a tumor suppressor by suppressing proto‐oncogene in many cancers.^9^ There have been reports on the role of FOXP3 in liver plasma but most of them focus on immune ways.^10^ As a result, it still remains a blank field of interaction between FOXP3 with microRNAs. Hence, we tried to connect miR‐198 with FOXP3 to see whether they are both involved in one pathway that related to liver neoplasm regulation.

MYC is a proto‐oncogene with great importance located on the long arm of chromosome 8, coding a nuclear protein that involved in nucleic acid metabolism and in mediating the cellular response to growth factors. Plenty evidence has shown that MYC plays a vital role in liver neoplasm proliferation and hepatocarcinogenesis.^11^ A number of microRNAs that related to MYC have been figured out, for example, miR‐203 for cutaneous squamous cell carcinoma 12 and especially, miR‐122,^13^ miR‐206,^4^ and miR‐214 14 for liver neoplasm. It has been proved that miR‐198 has the capacity to suppress liver cancer, and we decided to investigate the connection between miR‐198 with proto‐oncogene MYC to see if there is another mechanism behind the suppression caused by miR‐198.

Although similar studies have been performed to explore the connection between cancer, microRNAs, and cancer inhibitors, up to now there's few researches that combine FOXP3 with microRNAs or MYC, let alone the specific miR‐198, which means the underlying mechanism of the important tumor suppressor FOXP3 still needs exploring. In this study, we would like to figure out the relationship and the mechanism of FOXP3, miR‐198, and MYC in liver cancer. This study may offer new inspiration for developing brand new treatments for clinical use as well as potential biomarkers for diagnose and prognosis for liver neoplasm and help better understanding the pathology and regulation of liver neoplasm.

## 2 MATERIALS AND METHODS

### 2.1 Tissue samples

We obtained liver neoplasms and adjacent normal tissues (>3 cm laterally from the edge of the cancerous region) from 40 Chinese patients that received treatments in Hunan Provincial People's Hospital. All samples were confirmed by clinical and pathological diagnosis. All patients did not accept new adjuvant chemotherapy, new adjuvant endocrine therapy, or radiotherapy before the surgery. The inclusion and exclusion criteria are shown in Table S1. Patients’ clinical information is listed in Table 1. The tissues were immediately snap‐frozen in liquid nitrogen after surgical removal and stored at −80°C. All experiments were approved by the Ethics Committee in Hunan Provincial People's Hospital.

**Table 1:** Clinical and pathologic characteristics of 40 patients with liver cancer

Item	Characteristic	Data (n = 40)
Demographic	Gender [Male (%), Female (%)]	34 (85)/6 (15)
	Age (years, *x* ± *s*)	56 ± 8
Etiology	Hepatitis B	34 (85)
	Hepatitis C	3 (7.5)
	Other causes	3 (7.5)
Tumor grading	G1	21 (52.5)
	G2	13 (32.5)
	G3	6 (15)
AFP	>200 ng/mL	16 (40)
	5‐200 ng/mL	18 (45)
	<5 ng/mL	6 (15)
Tumor stage at diagnosis	BCLC A	8 (20)
	BCLC B	12 (30)
	BCLC C	20 (50)

AFP, alpha fetoprotein; BCLC, Barcelona‐Clí Liver Cancer.

### 2.2 Cell lines and cell culture

Two cell lines we used in this study were human liver neoplasm cell line HepG2 and human renal epithelial cell line 293T, both of them were purchased from GEFAN Biotechnology (Shanghai, China). Two cell lines were cultured in the high glucose Dulbecco's Modified Eagle's Medium (DMEM) (Cat No. 12491‐015, ThermoFisher Scientific, Waltham, MA, USA) which requires 10% Fetal Bovine Serum (FBS, Cat No. 10099‐141, ThermoFisher Scientific), a sodium bicarbonate buffer system (3.7 g/L) and a 5%‐10% CO_2_ environment to maintain a proper physiological hydrogen ion concentration (pH).

### 2.3 Quantitative real‐time polymerase chain reaction (qRT‐PCR)

Trizol reagent (Cat No. 15596026, Invitrogen, Carlsbad, CA, USA) was employed to isolate total RNA following the manufacturer's recommendations. The reverse transcription process was achieved using QuantiTect Reverse Transcription Kit (Cat No. 205310, QIAGEN, Dusseldorf, German), the real‐time PCR detection was finished by QuantiTect SYBR Green RT‐PCR Kit (Cat No. 204243, QIAGEN), all the processes were based on the protocols of instructions. The result was then analyzed using the 2^−△△Ct^ analysis method. The sequences of all primers are shown in Table 2.

**Table 2:** Primers for qRT‐PCR

Gene	Sequence	
MiR‐198	Forward	5′‐GGTCCAGAGGGGAGAT‐3′
	Reverse	5′‐GAATACCTCGGACCCTGC‐3′
MYC	Forward	5′‐TGGTCGCCCTCCTATGTTG‐3′
	Reverse	5′‐CCGGGTCGCAGATGAAACTC‐3′
FOXP3	Forward	5′‐GTGGCCCGGATGTGAGAAG‐3′
	Reverse	5′‐GGAGCCCTTGTCGGATGATG‐3′
GAPDH	Forward	5′‐AGCCACATCGCTCAGACAC‐3′
	Reverse	5′‐GAAGGTGAAGGTCGGAGTC‐3′
U6	Forward	5′‐CTCGCTTCGGCAGCACATA‐3′
	Reverse	5′‐AACGATTCACGAATTTGCGT‐3′

### 2.4 Recombinant plasmid construction and cell transfection

The sequences of si‐FOXP3 and si‐NC were obtained from BLOCK‐iTTM RNAi Designer (Invitrogen) which was shown in Table 3. The transfection processes were conducting under Lipofectamine^™^ 2000 (Cat No. 11668027, Invitrogen) following manufacturer's instructions. PcDNA3.1 FOXP3, pcDNA3.1 MYC (Cat No. ADV4), miR‐198 mimics (Cat No. B01001) and inhibitor (Cat No. B03001) in this study were all bought from GenePharma (Shanghai, China).

**Table 3:** Sequence of FOXP3 siRNA

Gene	Sequence (5′‐3′)
si‐FOXP3	TCTGGGCTCATAGGCACAT
si‐NC	TCTTCGTACGGAACGGCAT

### 2.5 Western blot

Cell lysates were abstracted using Thermo Scientific RIPA Buffer (Cat No. 89901) which contains 25 mmol/L Tris‐HCl (pH 7.6), 150 mmol/L NaCl, 1% Nonidet P 40 (NP‐40), 1% sodium deoxycholate, and 0.1% sodium dodecyl sulfate (SDS). Samples were centrifuged at 4°C, and the supernatants were collected. Quantified proteins were boiled with 5xloading buffer at 100°C for 5 minutes. Thirty micrograms protein was separated with 12% sodium dodecyl sulfate polyacrylamide gelelectrophoresis (SDS‐PAGE), and the separated protein was transferred to polyvinylidene fluoride (PVDF) membrane and then blocked in 5% nonfat milk medium under room temperature for about 2 hours. Primary antibodies Anti‐c‐Myc (1:1000, ab32072), Anti‐FOXP3 (1:1000, ab10901), Anti‐Bax (ab32503, 1:1000), Anti‐Bcl‐2 (1:500, ab59348), and Anti‐GAPDH (1:2500, ab9485) (Abcam, Cambridge, MA, USA) were added on the membranes and incubated overnight. After washed in Tris buffer saline Tween‐20 (TBST), HRP‐labeled goat anti‐rabbit secondary antibody IgG (1:2000, ab205718, Abcam) was added to the membranes, The mixture of primary and secondary antibodies was finally incubated for 1 hour and then washed with TBST for three times. Grayscale scanning was then used to visualize the immunoreactive proteins.

### 2.6 Cell Counting Kit 8 (CCK‐8) assay

After arriving at a density of 8000 cells/well, HepG2 cells in logarithmic phase were trypsinized and added into 96‐well plates for a 24‐hours preincubation. After 4‐hours incubation, 10 μL CCK8 solution was injected to each well. The cells at 0, 24, 48, and 72 hours were harvested using CCK‐8 kit (Cat No. C0037, Beyotime, Shanghai, China) according to the manufacturer's protocol. The absorption at different stages was recorded at 450 nm.

### 2.7 Colony formation

Digest the logarithmic phase transfected HepG2 cells with trypsin and seed them to 6‐well plates with a density of 1000 cells per well. Culture the cells at 37°C under 5% CO_2_ humidified atmosphere for 1‐2 weeks till the cells grow into visible colonies. The colonies were washed with PBS and fixed with 4% paraformaldehyde for 15 minutes. Discard the fixative and dye the cells with Gisma and observe the results under microscope after drying.

### 2.8 Flow cytometry (FCM) assay

Collect cells from each group and digest with 0.25% trypsin and seed them to 96‐well culture plates at the density of 20 000 cells per well. Two hundred microliters HEPES, 5 μL Annexin V/FITC, and 5 μL propidium iodide (PI) were added to each well, and cells were incubated in a dark room for 15 minutes at indoor temperature. FACS Calibur (BD Biosciences, San Jose, CA, USA) was used to perform the FCM assay. Those cells show positive for Annexin V FITC while negative for PI are apoptotic. Each assay was repeated independently for three times.

### 2.9 Chromatin Immunoprecipitation (ChIP) assay

The cells were immobilized in 1% formaldehyde, washed with PBS, and then suspended in SDS lysis buffer with proteinase inhibitor. The chromatin was sonicated to small sequences. The supernatant was collected after centrifuge, followed by standard ChIP analysis with antibody Anti‐FOXP3 (ab10901, Abcam). QRT‐PCR was performed to detect the abundance of relevant DNA. Primer sequences are provided in Table 4.

**Table 4:** Primers of miR‐198 promoter region sequence

Region	Forward sequence	Reverse sequence
−2000 ~ −1500	5′‐AATCTAGCCTGTGAACTGTC‐3′	5′‐CTGTGCCACCAATCCAAT‐3′
−1500 ~ −1250	5′‐GGAGGATAAGAATGCAAGGA‐3′	5′‐CACCAACCAACTCAGTAGG‐3′
−1250 ~ −1000	5′‐CTGTGTTCAGAGGCTTCC‐3′	5′‐ACAAAGGCAAGGGATGAC‐3′
−1000 ~ −750	5′‐ATCTGTAGACATCCTAAGAGC‐3′	5′‐CAAGCATATCTAACTGGTCAC‐3′
−750 ~ −500	5′‐TTATGTGCTGTTAGGTGCTA‐3′	5′‐CCTGCTTCTAATCAAGTCATC‐3′
−500 ~ −250	5′‐CCAGGCTTGAATCAATGC‐3′	5′‐AGGGAAGATGTCTGTTGTG‐3′
−250 ~ 0	5′‐CGGAGGTGCTCTCAATCAG‐3′	5′‐AGGTCGGTACTGGCTTAGG‐3′

### 2.10 Dual‐luciferase reporter gene assay

The promoter sequence of miR‐198 and the sequence of MYC were firstly amplified by PCR and then added to the carrier plasmid pmirGLO. Digest the 293T cells and transfect the cells using Lipofectamine 2000 kit (Cat No. 11668027, Invitrogen) according to the manufacturer's instructions. Each group (250 mL) was transfected with 30 μmol/L miR‐198 mimics/NC sequences and 6 μmol/L recombinant/blank plasmid. Assays were performed at 36 hours after transfection. Firefly luciferase activity was tested by recording the relative light unit (RLU) after adding the relevant reagent and followed by Renilla luciferase activity testing in similar way. The ratio of two RLU stands for the relative fluorescence intensity.

### 2.11 Statistical analysis

Statistical Product and Service Solutions (SPSS) 19.0 was applied through the whole of data analysis. The data were expressed as the mean ± standard deviation (SD). Statistics from more than two groups were examined using one‐way analysis of variance (ANOVA) for comparisons. For two‐sample comparisons, *t* test was used when the data were normal distributed while unpaired *t* test for not. Each experiment was repeated for more than three times (*P *<* *0.05 was considered statistically significance). GraphPad Prism 6.0 was used for drawing pictures.

## 3 RESULTS

### 3.1 FOXP3 inhibited MYC expression as well as tumor cell viability

**Figure 1:**
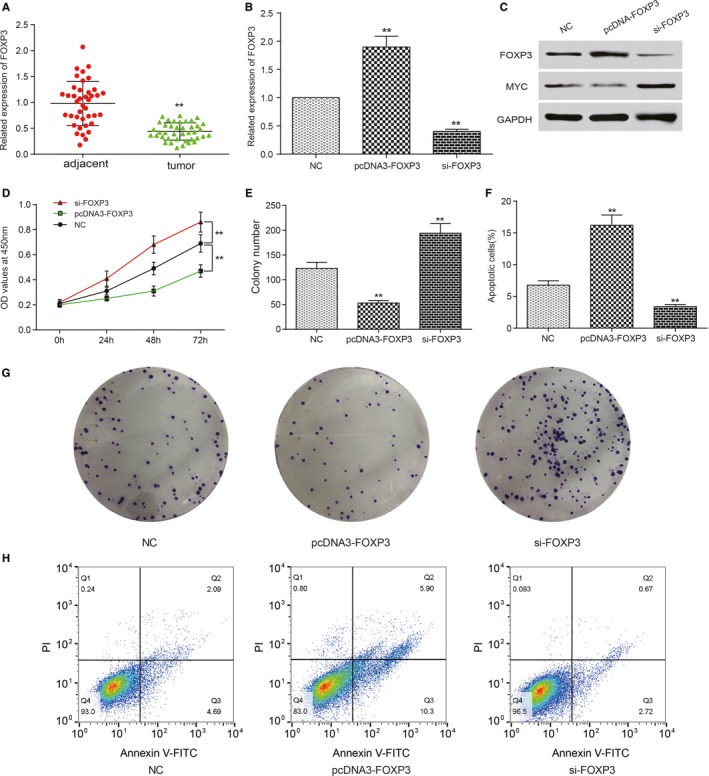
FOXP3 was a tumor suppressor in liver cancer. A, QRT‐PCT results showed that FOXP3 expressed significantly more in adjacent tissues than tumor tissues. **P <* *0.01, compared with adjacent group. B, HepG2 cells were transfected with pcDNA3.1, pcDNA3.1‐FOXP3, and si‐FOXP3, and qRT‐PCR was performed to examine the transfection efficiency. C, Western blot was used to determine the protein level of FOXP3 after transfection. D, CCK‐8 assay was used to access the effect of FOXP3 on cell viability. E and G, Colony formation assay was applied to test the proliferation ability of cells after transfection. F and H, FCM was used to evaluate the pro‐apoptotic effect of FOXP3 in HepG2 cells. **P <* *0.01, contrast to NC group

Firstly we performed qRT‐PCR to determine that whether FOXP3 was differentially expressed in adjacent tissues and tumor tissues, and the results showed that the expression level of FOXP3 mRNA in tumor tissues was much lower than that in adjacent tissues according to Figure 1A (*P *<* *0.01). The transfection efficiency was also verified by qRT‐PCR. Compared to NC group, there was remarkable increase on FOXP3 mRNA expression in pcDNA3.1‐FOXP3 group after transfection, while the FOXP3 level was obviously down‐regulated in si‐FOXP3 group (*P *<* *0.01, Figure 1B). We used Western blot to examination the expression of FOXP3 and MYC on protein level, and it turned out to be that high level of FOXP3 protein could attenuate the expression of MYC in HepG2 cells. And notably, MYC expression level was up‐regulated after knocking down FOXP3 with si‐RNA (Figure 1C). To determine the viability of liver neoplasm cells, we performed CCK‐8 assay and colony formation assay on NC cells and cells transfected with pcDNA3.1‐FOXP3 or si‐FOXP3. In comparison with NC group, cells in pcDNA‐FOXP3 group showed significant suppression on cell proliferation (*P *<* *0.01) but the si‐FOXP3 group turned out to be totally opposite (*P *<* *0.01, Figure 1D,E,G). Flow cytometry assay was also performed to test the effects of FOXP3 on cell apoptosis. We found that the apoptosis rate in pcDNA‐FOXP3 group was higher than that in NC group, while the apoptosis rate in si‐FOXP3 was the lowest among all the groups (Figure 1F,H). It suggested that high expression level of FOXP3 might contribute to cell apoptosis and inhibit cell proliferation.

### 3.2 Overexpression of FOXP3 enhanced the expression level of miR‐198 in HepG2 cells

**Figure 2:**
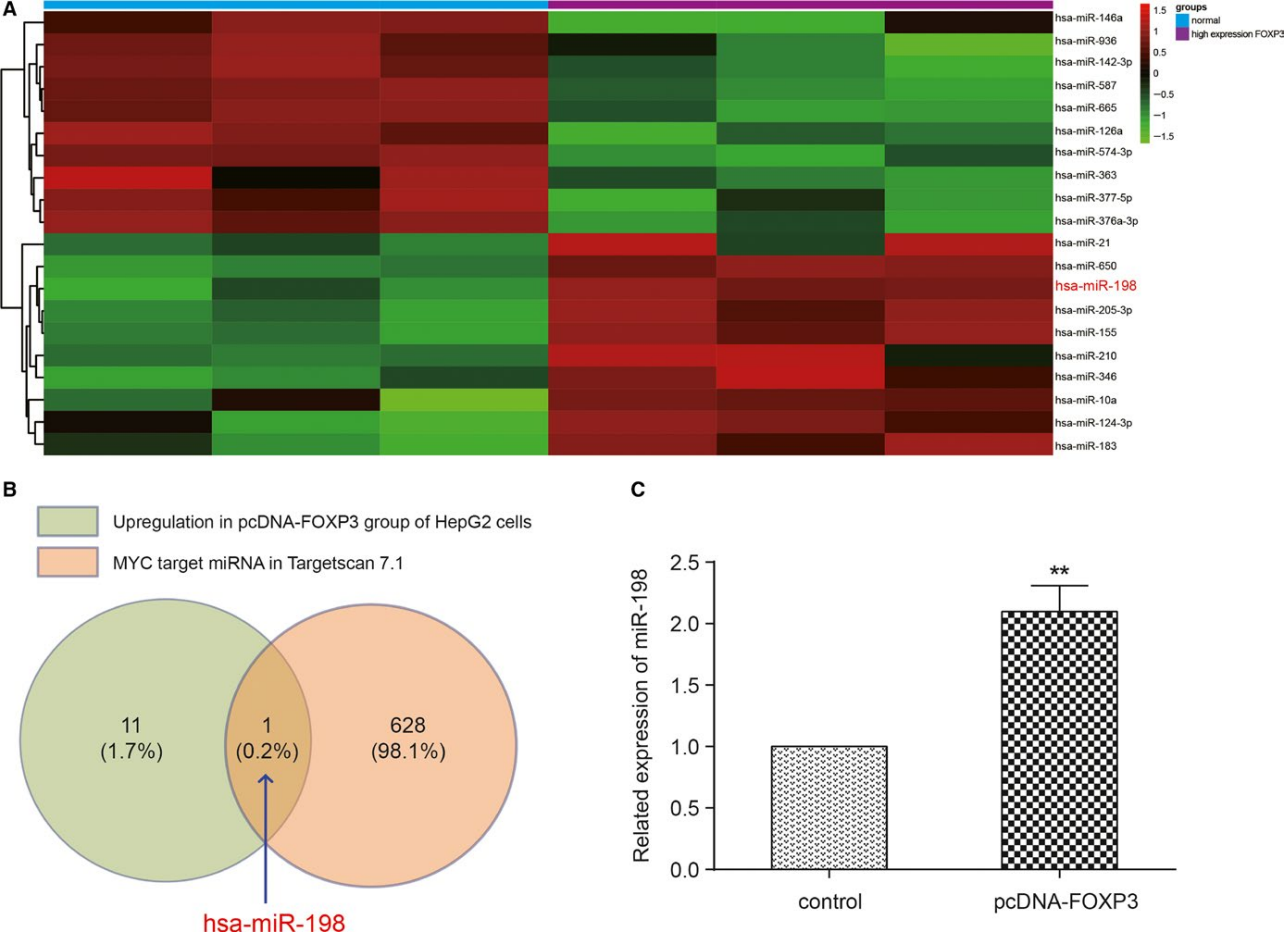
Overexpression of FOXP3 enhanced miR‐198 level. A, The heatmap of the top 10 up‐regulated or down‐regulated miRNAs after overexpression of FOXP3 in HepG2 cells. B, MiR‐198 was the only miRNA which both targeted at MYC and up‐regulated after FOXP3 overexpression. C, QRT‐PCR was performed to detect the expression level of miR‐198 in FOXP3 overexpression HepG2 cells. **P <* *0.01, compared with control group

We compared the miRNA levels in normal HepG2 cells and pcDNA‐FOXP3 cells using chip analyzing techniques and 28 miRNAs were screened out (Fold change>2, *P *<* *0.05). Among these miRNAs, 10 of them showed up‐regulation on expression in FOXP3 mRNA overexpression cells, while 18 were down‐regulated. We drew a heat map consist of 10 up‐regulated miRNAs and top 10 down‐regulated miRNAs (Figure 2A). Then we predicted the possible miRNA which may bind to MYC gene sequence using TargetScan7.1 software. We found that miR‐198 was the only one miRNA in the prediction group that showed up‐regulated expression in FOXP3 mRNA overexpression cells (Figure 2B). We performed qRT‐PCR again and found that the expression level of miR‐198 in FOXP3 overexpressed cells was 2.1 times of that in NC cells (Figure 2C).

### 3.3 FOXP3 had target relationship with miR‐198

**Figure 3:**
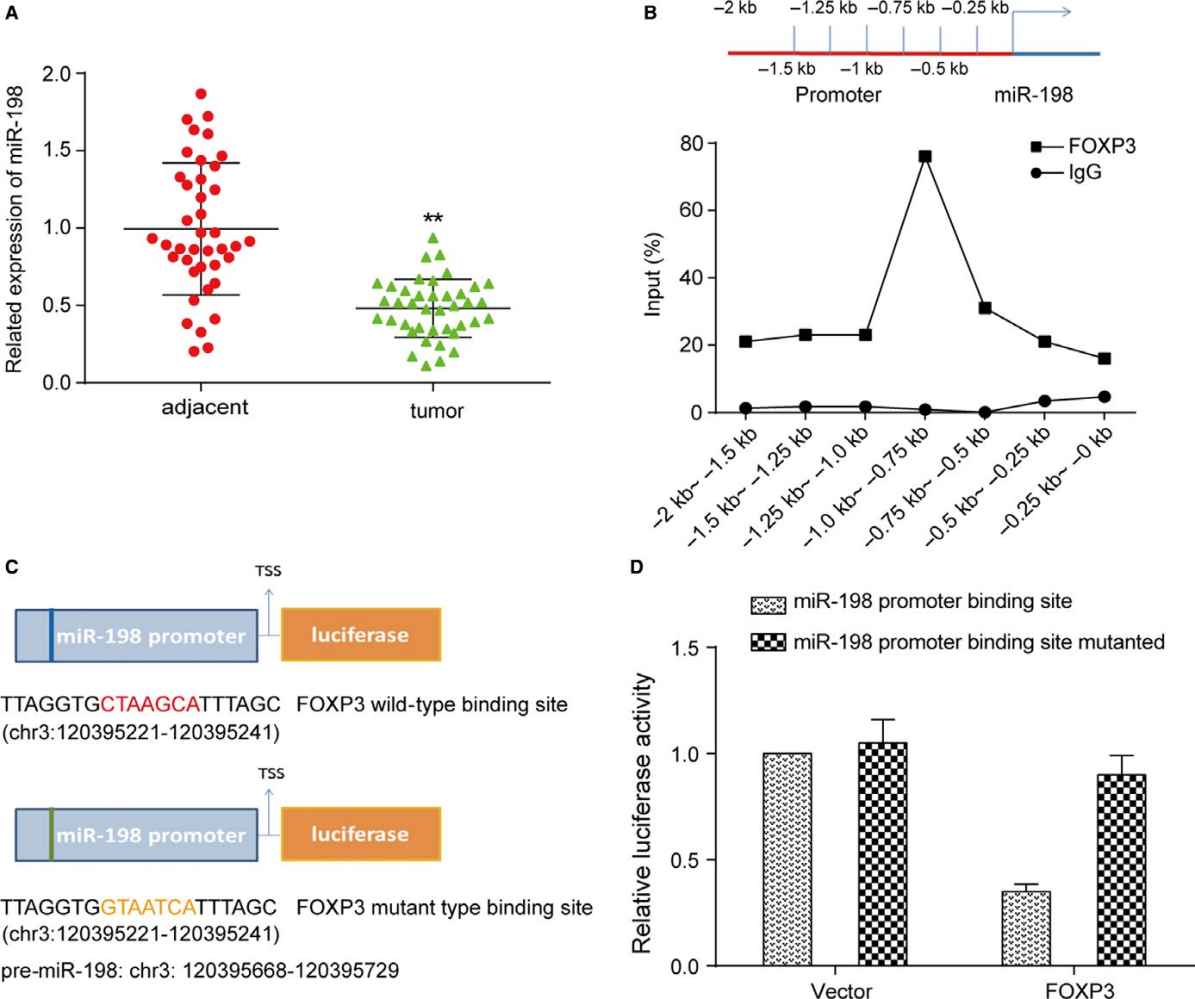
MiR‐198 was a target of FOXP3. A, MiR‐198 showed significant decline on expression level in tumor tissues in qRT‐PCR assay. **P <* *0.01, compared with adjacent group. B, ChIP assay revealed that FOXP3 regulated miR‐198 transcription by binding to its promoter sequence. C, Mutant promoter sequence was designed after predicting the possible bind site. The reporter gene was also added next to the promoter sequence. D, Dual‐luciferase reporter assay showed that FOXP3 had binding site with miR‐198. **P <* *0.01, compared with miR‐198 promoter binding site group

Thus, we further examined the connection between FOXP3 and miR‐198. To identify whether the consequence in cells was consistent with that in liver neoplasm tissues, qRT‐PCR was performed. From the result of qRT‐PCR for tissues, we figured out that miR‐198 expressed significantly lower in tumor cells than adjacent tissues (*P *<* *0.05, Figure 3A), which was another verification of previous experiments.

To locate the binding site of FOXP3 on miR‐198, we first use ChIP assay to tentatively find out the binding area. Under physiological conditions, the DNA in the cell is cross‐linked with the protein. After the chromatin was cut into small fragments (0.25 kb) by ultrasound treatment, the DNA fragment which bound to the target protein FOXP3 was precipitated by the specific recognition reaction of the antigen antibody. The result showed that the sequence at miR‐198 promoter −1.0 ~ −0.75 kb region combined most, which might be the most possible binding site for FOXP3 (Figure 3B), indicating that FOXP3 regulated the expression of miR‐198 by binding to the transcriptional start site. Then a dual‐luciferase reporter assay was performed to determine the exact binding position after transfection. We predicted the binding site and constructed plasmids containing mutant miR‐198 promoter sequence with reporter genes (Figure 3C). Both wild and mutant type plasmids were transfected together with FOXP3‐overexpressing plasmid or blank vector. If the transcription factor was capable of activating the target promoter, luciferase was expressed and its expression level was directly proportional to the intensity of action of the transcription factor. In FOXP3‐overexpressing group, the wild‐type group showed an obvious decline on fluorescence intensity comparing to the mutant group (*P *<* *0.01), while there was no significant variation in two blank vector groups (*P *>* *0.05, Figure 3D), which means that the mutant site at the promoter sequence was exactly the binding site of FOXP3 since the mutation blocked the inhibition of miR‐198 expression caused by FOXP3.

### 3.4 MYC is a potential target of miR‐198

**Figure 4:**
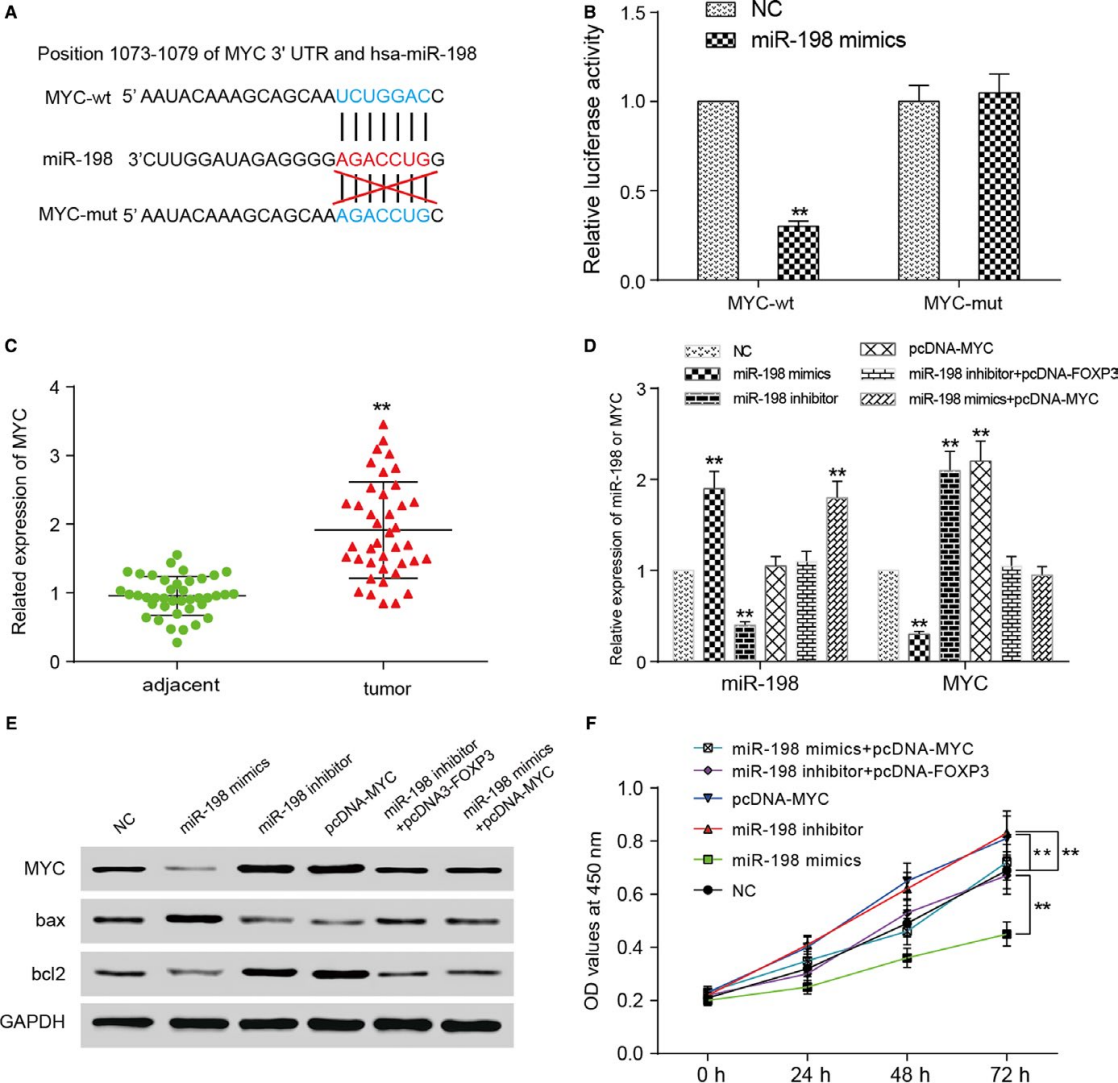
FOXP3 suppressed HepG2 cell proliferation by inhibiting MYC expression through miR‐198. A, Targetscan7.1 was used to predict that miR‐198 shared complementary sequences with MYC mRNA. B, The binding site between miR‐198 and MYC was verified by dual‐luciferase report assay. C, QRT‐PCR was carried out to examine the expression level of MYC in liver neoplasm tissues, showing that MYC expressed higher in tumor tissues than adjacent tissues. **P <* *0.01, in comparison with adjacent group. D, QRT‐PCR was used to examine the transfection efficiency in HepG2 by testing the transcriptional level of MYC and miR198. **P <* *0.01, compared with NC group. E, Western blot revealed the protein level of MYC and apoptosis‐related protein (bcl2 and bax) after transfection. F, Cells viability was tested by CCK‐8 assay, which indicated the carcinogenesis of MYC. **P <* *0.01, compared with NC group

To further verify the targeted relationship between miR‐198 and MYC, TargetScan7.1 was applied to predict the binding site on MYC and mutant sequences were designed (Figure 4A). The results of dual‐luciferase reporter assay showed declination on fluorescence intensity in wild‐type group after miR‐198 mimics transfection comparing to the blank group (*P *<* *0.01), but the mutant groups did not share the same feature (*P *>* *0.05, Figure 4B), which proved the predicted binding site. MYC expression was also verified to be highly expressed in tumor tissues (*P *<* *0.01, Figure 4C). For forward research the effects caused by miR‐198 expression on MYC and liver neoplasm cells, we transfected miR‐198 mimics, miR‐198 inhibitor, pcDNA3.1‐MYC, and pcDNA3.1‐FOXP3 into HepG2 cells and they were divided into 6 groups: NC group, miR‐198 mimics group, miR‐198 inhibitor group, pcDNA‐MYC group, miR‐198 inhibitor+pcDNA‐FOXP3 group, and miR‐198 mimics+pcDNA‐MYC group. The pcDNA‐MYC group was designed for observing MYC‐overexpression's influence on liver neoplasm cells, and miR‐198 inhibitor+pcDNA‐FOXP3 group as well as miR‐198 mimics+pcDNA‐MYC group was set up to restore the effects of miR‐198 and MYC. First qRT‐PCR was performed to test the transfection efficiency of each group by detecting the expression levels of miR‐198 and MYC (Figure 4D), showing down‐regulation of MYC expression in miR‐198 mimics group and up‐regulation in both miR‐198 inhibitor and pcDNA‐MYC groups, while no distinct change was noticed in miR‐198 inhibitor+pcDNA‐FOXP3 and miR‐198 mimics+pcDNA‐MYC groups, comparing to NC group. Western blot was carried out to test both MYC and apoptosis‐related proteins (bcl2 and bax) expressions. MYC protein level showed similar trend with qRT‐PCR results and bax/bcl2 ratio showed negative correlation with them (Figure 4E), suggesting that MYC protein suppressed cell apoptosis. CCK‐8 assay was then applied to test the viability of cells from each group. The OD values of different groups at same timepoint were compared with control group by paired Student's *t* test, and we found an outstanding decline of viability in miR‐198 mimics group contrast to NC group and obvious enhance in miR‐198 inhibitor group and pcDNA‐MYC group (*P *<* *0.01), with the rest two groups showing no evident difference with the control group (*P *>* *0.05, Figure 4F).

### 3.5 Effects of miR‐198 and MYC expression on cell proliferation and apoptosis

**Figure 5:**
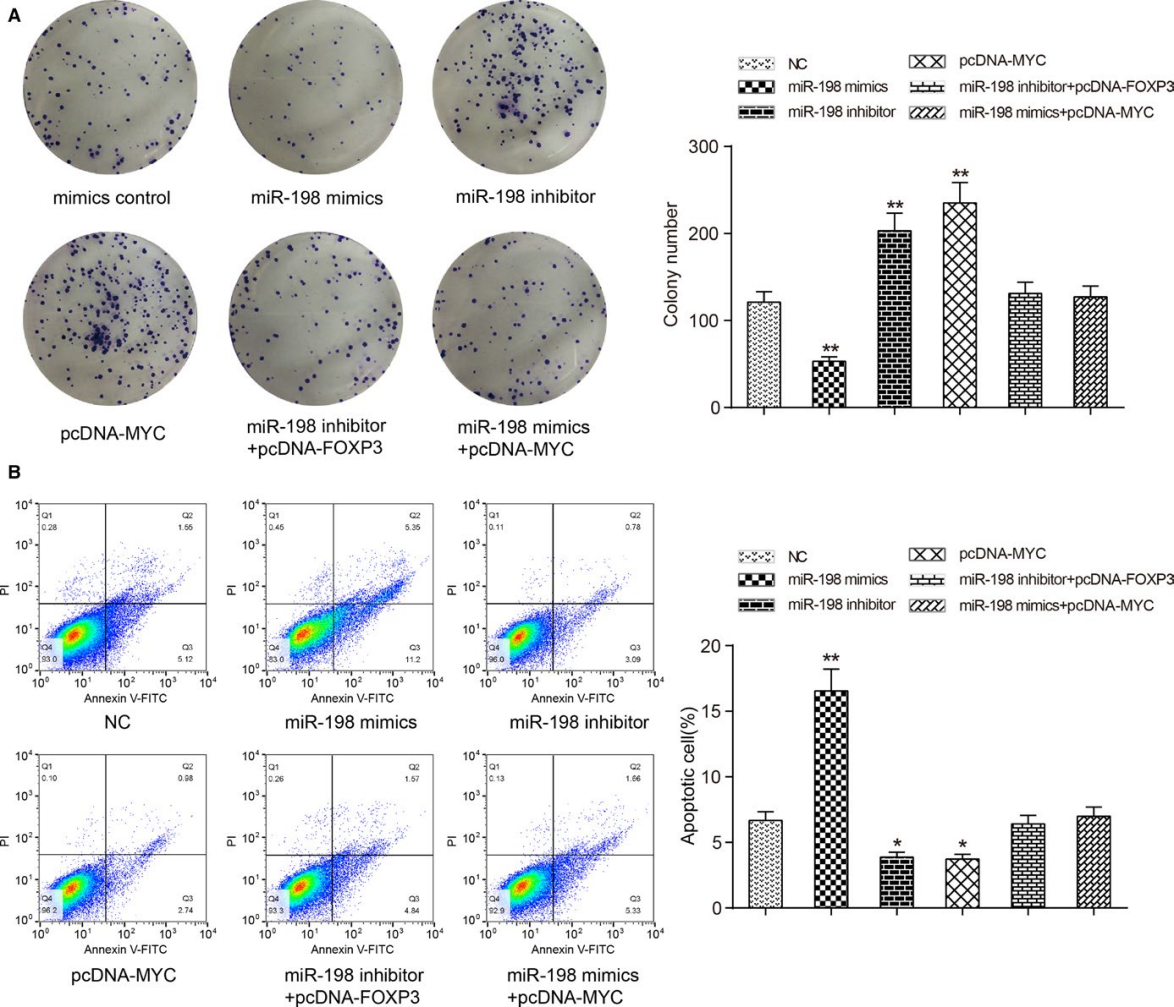
Effects of miR‐198 and MYC on HepG2 cell proliferation and apoptosis. A, Results of colony formation assay revealed that cell proliferation was suppressed by miR‐198 mimics, while enhanced by miR‐198 inhibitor as well as pcDNA‐MYC. There was no significant variation between miR‐198 inhibitor+pcDNA‐FOXP3 group or miR‐198 mimics+pcDNA‐MYC group with the NC group. B, Flow cytometry assay showed distinct increase on apoptosis rate in miR‐198 mimics group, while apoptosis rates in miR‐198 inhibitor group and pcDNA‐MYC group were slightly down‐regulated. MiR‐198 mimics+pcDNA‐MYC and miR‐198 inhibitor+pcDNA‐FOXP3 groups showed no difference compared with NC group. **P <* *0.01, compared with NC group

We next applied colony formation assay in liver neoplasm cells and it came out to be a significant down‐regulation on cell proliferation ability in miR‐198 mimics group (*P *<* *0.01), while that of cells from miR‐198 inhibitor group and pcDNA‐MYC group was strengthened (*P *<* *0.01). There showed no obvious change in inhibitor+pcDNA‐FOXP3 and miR‐198 mimics+pcDNA‐MYC groups comparing with NC group (*P *>* *0.05, Figure 5A). A significant increase on apoptosis rate in miR‐198 mimics group can be observed in the followed flow cytometry assay in comparison with NC group (*P *<* *0.01), but that in miR‐198 inhibitor and pcDNA‐MYC groups was slightly decreased by contrast (*P *<* *0.01). The two restore groups showed no evident variation with NC group (*P *>* *0.05, Figure 5B).

## 4 DISCUSSION

FOXP3 protein is a number of forkhead/winged helix families and has been recovered to function as suppresser of many cancers.^15^, ^16^, ^17^ Mir‐198 is a 22 bases RNA which regulates many proteins expression by interfering their translation.^6^, ^18^, ^19^, ^20^ But there are few reports on their cooperation for liver neoplasm. Also, the connection among miR‐198 or FOXP3 and the important proto‐oncogene MYC 21 has not been researched. This study further investigated the correlation between the MYC and FOXP3/miR‐198.

In our study, we examined the influence that FOXP3 had on MYC expression as well as the viability and the proliferation capacity of liver tumor cells in contrast with the adjacent tissues. An obvious decrease on MYC expression could be observed in FOXP3 mRNA overexpressing cells, while that in FOXP3 silenced cells was increased, indicating that FOXP3 inhibited MYC expression. Furthermore, overexpression of FOXP3 mRNA suppressed proliferation capacity and tended to have higher apoptosis rate, making it clear that FOXP3 strongly suppressed cells viability and promote cell apoptosis. Similar results are given by Huang et al^22^ that Quinacrine could accelerate U937 cell apoptosis by promoting the expression of apoptosis‐related protein bax via FOXP3 and miR‐183. However, FOXP3 mediated the expression of miR‐183 instead of promoting it in his study. Multiple evidences verify that FOXP3 have the capacity to regulate several miRNAs, positively or negatively.

Then, we tested the differentially expressed miRNAs in FOXP3 overexpressing cells and found miR‐198 was the only miRNA that might target MYC. The expression levels of both FOXP3 and miR‐198 were both lower in tumor tissues, and subsequent experiments proved that FOXP3 could lead to the rise of miR‐198 levels. Notably, Murakami et al^23^ reported a decrease of miR‐198 when liver metastasis occurred in colorectal cancer. It remained suspense that whether decrease of miR‐198 caused tumor metastasis or metastasis led to the decrease of miR‐198. We suppose that miR‐198 regulation pathway is a key to cancer treatment and appeal for more attention on it. In addition, due to the strong association of FOXP3/miR‐198 levels with cancer status, they may have the potential as new biomarkers for clinical diagnose and prognosis.

Finally we confirmed the direct target relationship among FOXP3 with miR‐198 and miR‐198 with MYC, the binding site for each was also accurately located meanwhile that FOXP3 bound to the promoter of miR‐198 while miR‐198 bound to the 3′‐UTR of MYC mRNA, filling the blank of this area. MiR‐198 had already been confirmed to have suppression on tumor cells by targeting serine hydroxymethyltransferase 1 (SHMT1).^24^ However, in our study the proto‐oncogene MYC was the target rather than an enzyme gene, thus providing a new perspective on miR‐198‐related cancer inhibition. Besides, this finding radically proved the interactions between these 3 molecules and uncovered the mechanism of this regulation pathway, providing a new entry point for intervening the regulation function. Since miR‐198/FOXP3 has a strong suppression on tumor cells, more powerful alternative inhibitors that direct at these two sites could be developed to better inhibit the proto‐oncogene MYC expression for clinical liver neoplasm treatment. This study may also be combined with previous findings to develop multipurpose medicine that targets several cancer‐related gene/RNA/protein for better curative effects.^25^, ^26^

However, there are some limitations in our study. A previous study has revealed that FOXP3 is able to regulate c‐Myc directly,^27^ which may be another mechanism of FOXP3 and need further study. On the other hand, FOXP3 may also influence liver cancer through other target genes, which are deserved to explore as well. Since FOXP3, miR‐198 and MYC do not only exist in liver cells, whether this study could be repeated in other types of cancers remains unclear. If not, there might be more unknown elements that involve in this regulation pathway.

In summary, FOXP3 is confirmed to have the capacity to suppress proto‐oncogene MYC in liver neoplasm by up‐regulating miR‐198 through binding to its promoter, while miR‐198 targets the 3′‐UTR of MYC mRNA to inhibit its translation. The high expression of FOXP3 and, especially miR‐198, is verified to have strong suppression on liver tumor cells viability and proliferation, while promoted the apoptosis meanwhile. This study may offer new potential biomarkers as well as new entry points for cancer inhibitor development for clinical use.

## CONFLICT OF INTEREST

None declared.
