# Supplementary material for: FOXP3 inhibits MYC expression via regulating miR‐198 and influences cell viability, proliferation and cell apoptosis in HepG2
Source: Cancer Med. 2018 Oct 30;7(12):6182–92. doi: 10.1002/cam4.1780 (PMC6308052; doi:10.1002/cam4.1780)
Supplement: Supplementary file 1 [file CAM4-7-6182-s001.docx]

**Table S1. Inclusion and exclusion criteria for tissue sampling**

**Inclusion criteria**

Only patients who met the following inclusion criteria were enrolled:

1. At least 18 years of age and signed written informed consent.

2. Confirmed by clinical and pathological diagnosis according to The National Comprehensive Cancer Network (NCCN).

3. No previous palliative chemotherapy or radiotherapy.

4. Tumor size > 4 cm.

5. Life expectancy of at least 3 months.

**Exclusion criteria**

Patients were excluded from the study if they had one or more of the following:

1. Lost to follow-up.

2. Incomplete results of relevant clinical data examination.

3. Metastatic hepatic cancer.

4. With major underlying diseases or other cancers.

5. Pregnancy or lactation.

6. Current alcohol or drug addiction.
